# Placental growth factor inhibition modulates the interplay between hypoxia and unfolded protein response in hepatocellular carcinoma

**DOI:** 10.1186/s12885-015-1990-6

**Published:** 2016-01-11

**Authors:** Yves-Paul Vandewynckel, Debby Laukens, Lindsey Devisscher, Eliene Bogaerts, Annelies Paridaens, Anja Van den Bussche, Sarah Raevens, Xavier Verhelst, Christophe Van Steenkiste, Bart Jonckx, Louis Libbrecht, Anja Geerts, Peter Carmeliet, Hans Van Vlierberghe

**Affiliations:** Department of Hepatology and Gastroenterology, Ghent University Hospital, De Pintelaan 185, 1K12IE, B-9000 Ghent, Belgium; ThromboGenics NV, Heverlee, Belgium; Department of Pathology, Ghent University Hospital, Ghent, Belgium; Laboratory of Angiogenesis and Neurovascular link, Vesalius Research Centre, KU Leuven, Leuven, Belgium; Laboratory of Angiogenesis & Neurovascular Link, Vesalius Research Centre, VIB, Leuven, Belgium

**Keywords:** Carcinoma, Hepatocellular, Placenta growth factor, Tumor Microenvironment, Unfolded protein response, Cell hypoxia, Angiogenesis Modulating Agents, Hep G2 cells

## Abstract

**Background:**

Hepatocellular carcinoma (HCC) is a leading cause of cancer-related mortality. We previously showed that the inhibition of placental growth factor (PlGF) exerts antitumour effects and induces vessel normalisation, possibly reducing hypoxia. However, the exact mechanism underlying these effects remains unclear. Because hypoxia and endoplasmic reticulum stress, which activates the unfolded protein response (UPR), have been implicated in HCC progression, we assessed the interactions between PlGF and these microenvironmental stresses.

**Methods:**

PlGF knockout mice and validated monoclonal anti-PlGF antibodies were used in a diethylnitrosamine-induced mouse model for HCC. We examined the interactions among hypoxia, UPR activation and PlGF induction in HCC cells.

**Results:**

Both the genetic and pharmacological inhibitions of PlGF reduced the chaperone levels and the activation of the PKR-like endoplasmic reticulum kinase (PERK) pathway of the UPR in diethylnitrosamine-induced HCC. Furthermore, we identified that tumour hypoxia was attenuated, as shown by reduced pimonidazole binding. Interestingly, hypoxic exposure markedly activated the PERK pathway in HCC cells *in vitro*, suggesting that PlGF inhibition may diminish PERK activation by improving oxygen delivery. We also found that PlGF expression is upregulated by different chemical UPR inducers via activation of the inositol-requiring enzyme 1 pathway in HCC cells.

**Conclusions:**

PlGF inhibition attenuates PERK activation, likely by tempering hypoxia in HCC via vessel normalisation. The UPR, in turn, is able to regulate PlGF expression, suggesting the existence of a feedback mechanism for hypoxia-mediated UPR that promotes the expression of the angiogenic factor PlGF. These findings have important implications for our understanding of the effect of therapies normalising tumour vasculature.

**Electronic supplementary material:**

The online version of this article (doi:10.1186/s12885-015-1990-6) contains supplementary material, which is available to authorized users.

## Background

Hepatocellular carcinoma (HCC) ranks as the second leading cause of cancer-related mortality worldwide [1]. Conventional chemotherapy is ineffective, and targeted therapy for advanced HCC with sorafenib, which targets Raf and platelet-derived and vascular endothelial growth factor (VEGF) receptor tyrosine kinase signalling, shows only a limited survival benefit [2].

The VEGF signalling pathways play central roles in angiogenesis [3]. VEGF-A binds to two tyrosine kinase receptors, VEGFR-1 and VEGFR-2. Most of the biological effects of VEGF-A are mediated by VEGFR-2 [3]. The placental growth factor (PlGF, four isoforms: PlGF-1-4) binds to VEGFR-1 and induces responses in endothelial, malignant, and immune cells [4]. VEGFR-1 has weak tyrosine kinase activity but a substantially higher binding affinity for VEGF-A than VEGFR-2. Although VEGFR-1 may act as a trap for VEGF-A, it also transmits signals in response to PlGF via its tyrosine kinase domains [4, 5]. A role for VEGFR-1 during tumour angiogenesis has been suggested [5, 6]. VEGFR-1 expression in HCC tissues is higher than that in peritumoural tissues and correlates with worse survival after resection [7, 8].

Importantly, genetic or pharmacological inhibition of PlGF reduces tumour growth and induces vessel normalisation in different preclinical models, including the diethylnitrosamine-induced HCC model [5, 9, 10]. Although anti-PlGF antibodies are controversial [11], evidence for the dose and specificity of the anti-PlGF-2 antibody clone 5D11D4 was previously provided [5]. Furthermore, disease stabilization for 12 months has been observed with anti-human PlGF monoclonal antibody TB403 in 2 out of 23 patients with advanced solid tumours refractory to standard therapy, confirming the need for a better understanding of the effect of PlGF inhibition on tumour biology [12].

The endoplasmic reticulum (ER) consists of a membranous network in which proteins are synthesised, post-translationally modified and folded. Therefore, the lumen houses chaperones, including protein disulfide isomerase A4 (PDIA4), calnexin (CANX), glucose-regulated protein-78 (GRP78) and −94 (GRP94) [12–14]. Several perturbations in the protein folding, such as hypoxia, glucose deprivation and oxidative stress, lead to the accumulation of unfolded proteins in the ER, a phenomenon called ER stress. ER stress triggers the unfolded protein response (UPR), which leads to an adaptive transcriptional response involved in protein quality control, redox homeostasis and angiogenesis. Paradoxically, the UPR also coordinates pro-apoptotic responses to ER stress [13, 14]. Interestingly, ER stress is present in human and experimental HCC, and modulating the UPR could hold important therapeutic potential [15, 16].

Three major ER stress sensors have been identified, as follows: PKR-like ER kinase (PERK), inositol-requiring enzyme 1 (IRE1) and activating transcription factor 6 (ATF6) [13]. The effect of ATF6 on cell fate is primarily cytoprotective, whereas the effect of IRE1 and PERK is presumed to be both pro-adaptive and pro-apoptotic [13, 14, 17]. However, inhibition of the PERK pathway induces antitumour effects in experimental HCC [14]. Following the release of GRP78, PERK phosphorylates the eukaryotic initiation factor 2α (eIF2α), leading to the attenuation of global translation. However, the translation of certain transcripts, such as activating transcription factor 4 (*ATF4*), is favoured. ATF4 induces genes involved in protein quality control, amino acid biosynthesis and the induction of apoptosis via C/EBP homologous protein (*CHOP*) [13]. IRE1 activation results in X-box-binding protein 1 (*XBP1*) mRNA splicing to generate a more active spliced XBP1 (XBP1s), which induces the genes involved in protein folding, such as endoplasmic reticulum DnaJ homolog 4 (*ERDJ4*) and *CANX* [18]. ATF6 is mobilised to the Golgi, where it is cleaved, releasing a transcriptionally active fragment, which in turn induces the expression of homocysteine-responsive ER-resident ubiquitin-like domain member 1 (HERPUD1), unspliced XBP1 (XBP1u) and chaperones including PDIA4 [12, 17].

In this study, we investigated whether vessel normalisation induced by PlGF blockade modulates the activation of the UPR or oxygen levels in experimental HCC and whether PlGF expression is regulated by ER stress. Collectively, we revealed that PlGF inhibition reduced hypoxia and the activation of the PERK pathway of the UPR in the tumour nodules of the carcinogen-induced mouse model. Furthermore, PlGF expression was upregulated by divergent ER stress stimuli *in vitro*. These results provide important insight into the reciprocal interactions between PlGF and the tumour microenvironment.

## Methods

### Animals

Wild type 129S2/SvPasCrl mice were purchased from Charles River (Belgium), and PlGF^−/−^ knockout (PlGFKO) 129S2/SvPasCrl mice were obtained from the laboratory of Angiogenesis & Neurovascular link (Leuven, Belgium). Both were maintained as previously described [5]. All mice were genotyped by PCR before the start of the experiments. PlGF-deficient mice are born at normal Mendelian ratios and do not show any obvious vascular anomalities [19]. Five-week-old males received weekly intraperitoneal saline or diethylnitrosamine (DEN) (35 mg/kg, in saline) injections [20]. A murine anti-PlGF monoclonal antibody (validated clone 5D11D4 [5]; referred to as aPlGF) was obtained from Thrombogenics (Leuven, Belgium). Wild type mice that received DEN for 25 weeks were subsequently treated for 5 weeks with aPlGF (intraperitoneally, 25 mg/kg; 2x/week) or IgG (same regimen, *n* = 10 in each group). Wild type mice that received saline for 25 weeks were subsequently treated for 5 weeks with aPlGF (same regimen) or IgG (same regimen, *n* = 10 in each group). Pimonidazole HCl (Hypoxyprobe-1 Inc., Burlington, MA, USA) was intraperitoneally administered to 4 random mice per group in a single dose of 60 mg/kg one hour before sacrifice. Male PlGFKO mice and their wild type littermates received DEN for 30 weeks (*n* = 12 in each group). After 30 weeks, blood was collected from the retro-orbital sinus under isoflurane anaesthesia. After macroscopic evaluation and the quantification of the number of hepatic tumours with a minimum diameter of 2 mm, the livers were fixed in 4 % phosphate-buffered formaldehyde (Klinipath) and embedded in paraffin or snap frozen in liquid nitrogen. Tumour nodules were isolated by microdissection (Carl Zeiss, Bernreid, Germany) for expression analysis. Haematoxylin/eosin and reticulin staining were performed to assess the tumour burden, and the results were assessed by 2 independent observers. All protocols were approved by the Ethical Committee of experimental animals at the Faculty of Health Sciences, Ghent University, Belgium (ECD 11/52).

### Cell culture

HepG2 (HB-8065; ATCC, Virginia, USA), Hep3B (HB-8064; ATCC) and Huh7 (kindly provided by Dr. Olivier Govaere (University of Leuven, Belgium)) cells were cultured in DMEM supplemented with 10 % foetal bovine serum (Life Technologies, Ghent, Belgium). None of the three cell lines used require ethical approval. Cells were incubated for 24 h or 48 h with a PERK inhibitor (0.3 μM; GSK2656157, NoVi Biotechnology, Shandong, China), an IRE1 inhibitor (8 μM; 4μ8C, Calbiochem, Massachusetts, USA), tauroursodeoxycholic acid (TUDCA, 1 mM), tunicamycin (1 μM), thapsigargin (150 nM) or quercetin (100–300 μM) and compared to equal volumes of solvent. All reagents were obtained from Sigma (Diegem, Belgium) unless stated otherwise. Hypoxic atmosphere (1 % oxygen) was established in a hypoxic chamber (AnaeroGen; Oxoid, Basingstoke, UK). Experiments were carried out in quadruplicate and independently repeated three times.

Detailed information regarding total RNA extraction, quantitative real-time PCR, Western blotting, and immunohistochemistry is provided in the Additional file 1.

### Statistics

Statistical analyses were performed using SPSS 21 (SPSS, Chicago, USA). Values are presented as the means ± SD or fold change relative to the mean expression in controls. Kolmogorov-Smirnov test was used to test for normality. Normally distributed data were subjected to the unpaired Student’s t-tests. Multiple groups were compared by one-way analysis of variance (ANOVA) with Bonferroni correction. Non-normally distributed data were tested using the Mann–Whitney U-test. Two-tailed probabilities were calculated; a p-value less than 0.05 was considered statistically significant.

## Results

### PlGF inhibition induces antitumour effects and vessel normalisation in experimental HCC

First, we validated the previously reported antitumour effects and vessel normalisation induced by PlGF blockage [5, 9]. When wild type mice with established HCC were treated with aPlGF (*n* = 10) or IgG (*n* = 10) from 25 weeks onward for 5 weeks, 20 % of mice receiving control IgG died, whereas only 10 % died in the aPlGF group. Additionally, aPlGF-treated mice developed fewer nodules per liver (all sizes: 17.6 ± 4.9 after IgG versus 12.7 ± 3.2 after aPlGF; *p* < 0.05). After 30 weeks of DEN administration to wild type (*n* = 12) or PlGFKO (*n* = 12) mice, 25 % of wild type mice compared to 16 % of PlGFKO mice succumbed, and fewer tumour nodules per liver were observed in PlGFKO mice (22.4 ± 4.8 in wild type versus 15.8 ± 6.2 in PlGFKO; *p* < 0.05). Furthermore, several capillaries in control HCC nodules had an abnormal shape and size (Additional file 2: Figure S1A). In PlGF-blocked tumours, fewer capillaries, as shown by endoglin staining, were tortuous (aPlGF: *p* < 0.05 and PlGFKO: *p* < 0.01; Additional file 2: Figure S1B). These results confirm that PlGF blockage induces antitumour effects and partially normalises the abnormal tumour vessel structure.

### PlGF inhibition reduced chaperone expression and activation of the Perk pathway in experimental HCC

**Fig. 1:**
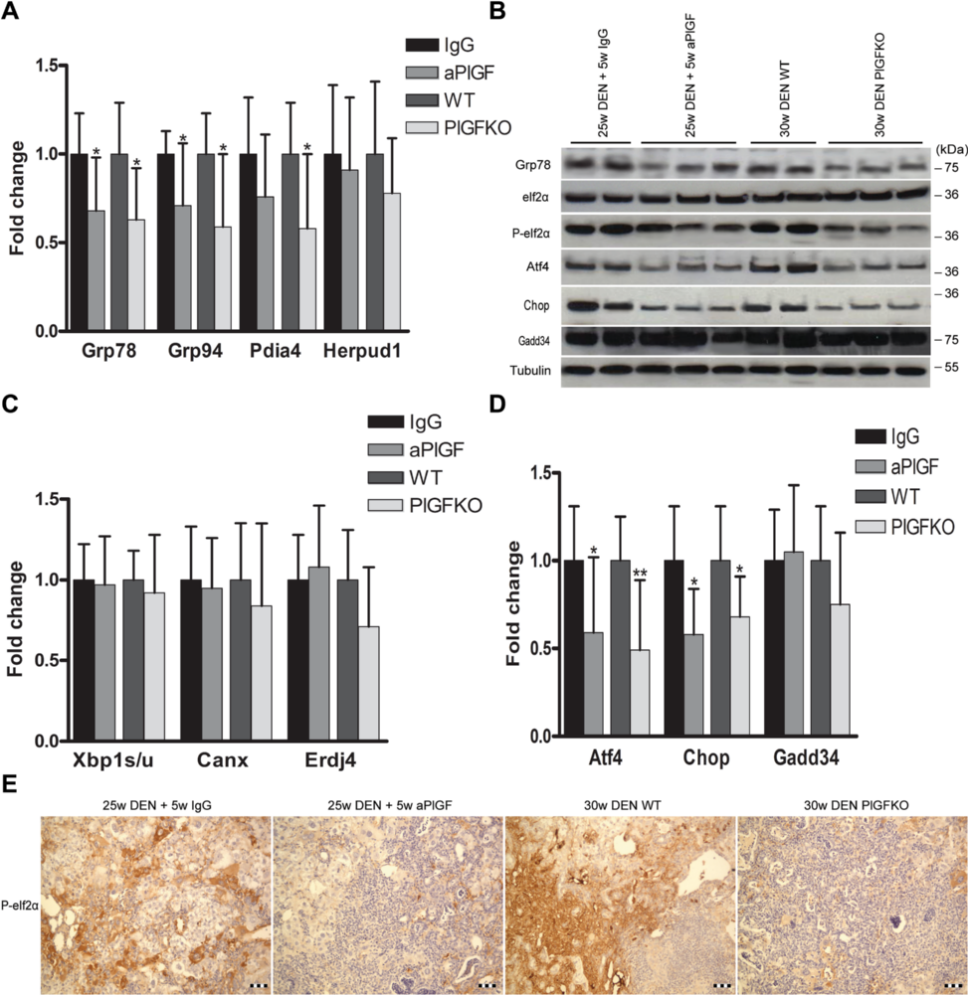
PlGF inhibition tempers the activation of the UPR in an orthotopic mouse model of HCC. **a** Quantitative real-time PCR analysis of the ER chaperones *Grp78*, *Grp94* and *Pdia4* and *Herpud1* in aPlGF-treated and PlGFKO mice. Relative fold changes were calculated using the ΔΔCT method. **b** Immunoblotting for UPR-mediated proteins. **c** Quantitative real-time PCR analysis of ER chaperones of Ire1-mediated splicing of *Xbp1* and Ire1 targets *Canx* and *Erdj4*, **d** Perk-related genes *Atf4*, *Chop* and *Gadd34*. *p < 0.05, **p < 0.01, ***p < 0.001. IgG = 25w DEN + 5w IgG, aPlGF = 25w DEN + 5w aPlGF, WT = 30w DEN in wild type (WT) mice, PlGFKO = 30w DEN in PlGF^−/−^ knockout mice. **e** Immunostaining for phospho-eIf2α in mouse livers following the indicated treatment. Scale bars: 100 μm

We among others previously described the UPR pattern in DEN-induced HCC [15]. Here, we evaluated the effect of PlGF inhibition on this pattern in isolated tumours. The administration of aPlGF downregulated the mRNA expression of the ER stress-induced chaperones Grp78 and Grp94 in the tumours, compared to the IgG group (*p* < 0.05; Fig. 1a). Additionally, the PlGFKO mice that received DEN for 30 weeks showed reduced levels of Grp78 (*p* < 0.05) and Grp94 (*p* < 0.05) in the tumours compared to their wild type littermates. Western blotting demonstrated reduced protein expression of Grp78 in the tumours of the aPlGF-treated and PlGFKO mice compared to those of the IgG-treated and wild type control group, respectively (Fig. 1b).

The Ire1-mediated splicing of Xbp1 was unaltered by PlGF inhibition (Fig. 1c). Accordingly, the targets of Xbp1s, Canx and Erdj4, showed a similar expression level compared to the corresponding controls.

Western blot analysis (Fig. 1b and Additional file 3: Figure S2) and immunostaining (Fig. 1e) showed that the Perk-mediated phosphorylation of eIf2α was reduced in the HCC tissues of aPlGF-treated and PlGFKO mice compared to IgG-treated and wild type controls resp.. Atf4 mRNA (aPlGF: *p* < 0.05 and PlGFKO: *p* < 0.01; Fig. 1d) and protein (Fig. 1b) expression in the nodules were decreased by PlGF inhibition. Further, Chop mRNA (*p* < 0.05; Fig. 1d) and protein (Fig. 1b) levels were decreased. Next, we assessed the expression of Growth arrest and DNA damage-inducible protein (Gadd34), which initiates eIf2α dephosphorylation leading to a negative feedback loop of the Perk pathway [13]. Gadd34 levels were unaltered (Fig. 1b and d), indicating that PlGF inhibition did not enhance this negative feedback loop. Also, the mRNA and protein levels of the UPR sensor Perk itself were unaltered, excluding a direct effect of PlGF on Perk expression (Additional file 4: Figure S3A-B). Overall, these data indicate that PlGF inhibition indirectly diminished Perk signalling in HCC.

To examine the Atf6 pathway, Pdia4 and Herpud1 mRNA expression was monitored (Fig. 1a). Only Pdia4 mRNA was downregulated in the tumours of PlGFKO mice compared to their wild type littermates (*p* < 0.05).

Importantly, wild type mice that received saline for 25 weeks and were subsequently treated with aPlGF for 5 weeks demonstrated no significant differences in the hepatic mRNA expression of the selected UPR targets compared to those receiving control IgG treatment (data not shown). Thus, these results demonstrate that PlGF inhibition reduces the intratumour expression of chaperones, such as Grp78, Grp94 and Pdia4, as well as the activation of the Perk pathway.

### PlGF inhibition reduces intratumour hypoxia

**Fig. 2:**
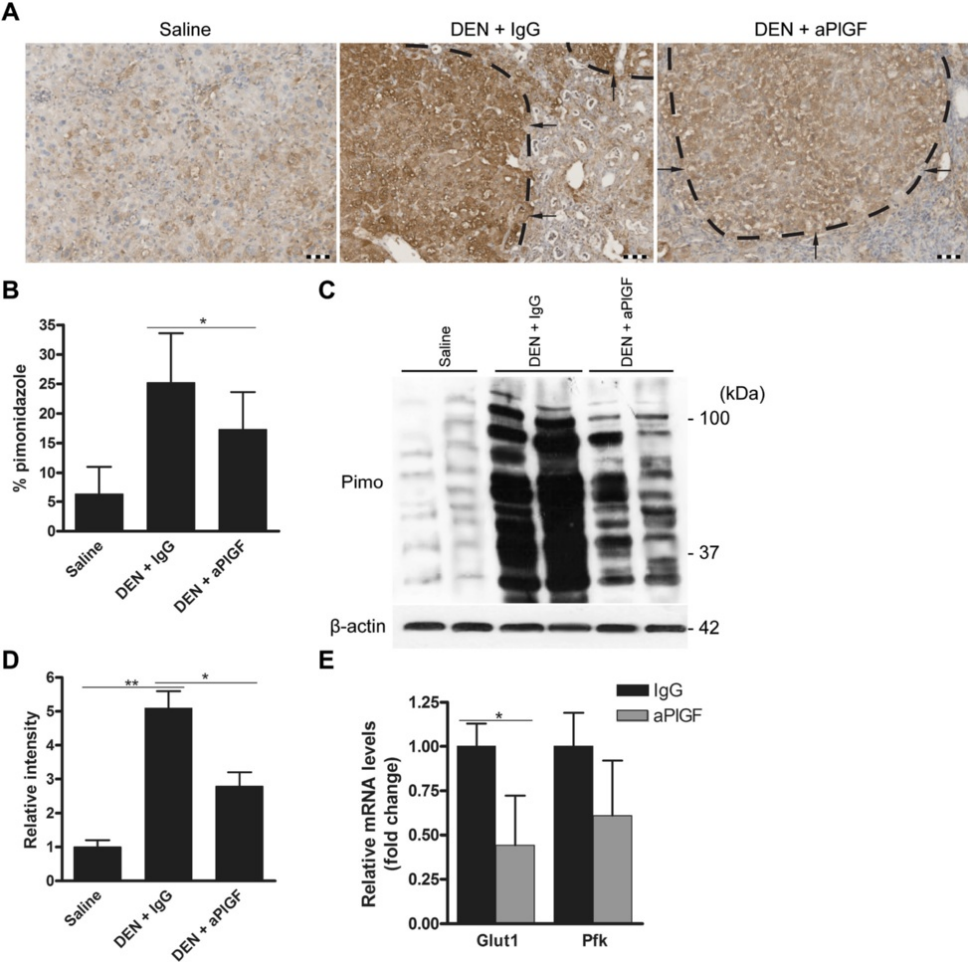
PlGF inhibition reduces intratumour hypoxia in experimental HCC. **a** Immunostaining for pimonidazole in mouse livers following the indicated treatment. Arrows indicate tumours. Scale bars: 100 μm. **b** Quantification of the immunostaining for pimonidazole. **c** Lysates of control liver tissue or isolated DEN-induced tumours were subjected to Western blotting for detection of pimonidazole adducts (Pimo). Blotting of β-actin is shown as a loading control. **d** Densitometry analysis of the pimonidazole blot in (**c**). **e** Real-time PCR analysis of *Glut1* and *Pfk* mRNA levels in tumour tissues. IgG = 25w DEN + 5w IgG, aPlGF = 25w DEN + 5w aPlGF. Data are presented as the means ± SD. *p < 0.05

We previously showed that PlGF inhibition induces vessel normalisation (Additional file 2: Figure S1; [5, 21]). To investigate whether these vascular changes were functionally relevant or, in other words, whether PlGF inhibition effectively increased the oxygen levels in the hepatic tumours of the used mouse model, we applied pimonidazole, a molecule that binds only hypoxic areas *in vivo* and can be detected after sacrifice by immunohistochemistry (Fig. 2a). Indeed, administration of aPlGF significantly reduced tumoural pimonidazole binding (*p* < 0.05; Fig. 2a-b). To improve the quantification method of the binding of pimonidazole, Western blotting for detection of pimonidazole adducts in isolated DEN-induced tumours was performed (Fig. 2c). Densitometry analysis confirmed that the liver tumours were characterized by increased pimonidazole binding and that administration of aPlGF reduced pimonidazole binding in the tumours (*p* < 0.05; Fig. 2c-d). Finally, aPlGF downregulated the expression of hypoxia-inducible genes *Glut1* (*p* < 0.05) and *Pfk* (*p* = 0.07) in the DEN-induced HCC nodules (Fig. 2e). Thus, aPlGF effectively tempered the induction of tumour hypoxia.

### Hypoxia activates the PERK pathway

**Fig. 3:**
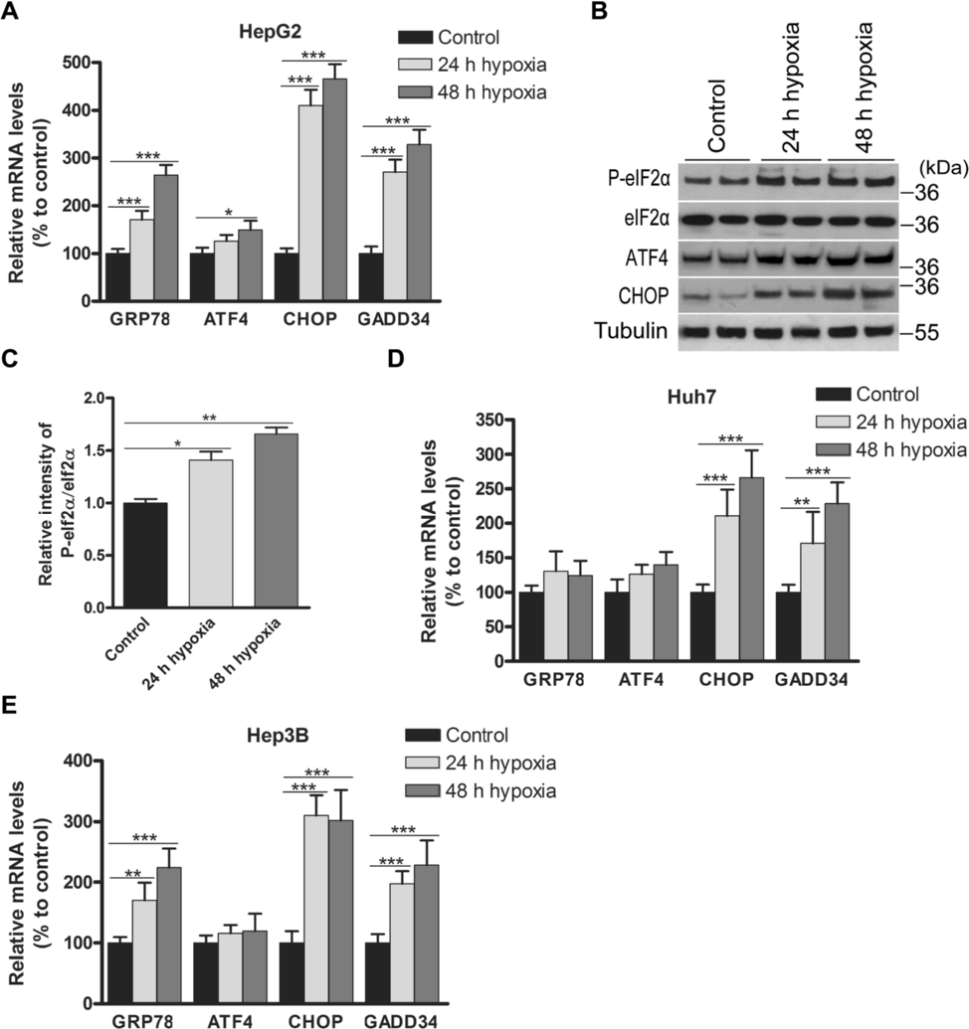
Hypoxia activates the PERK pathway in HCC cells. **a** HepG2 cells were cultured in normoxia or hypoxia for 24 h or 48 h. The PERK targets, *GRP78*, *ATF4*, *CHOP* and *GADD34* mRNA were detected by Real-time PCR analysis. **b** Expressions of phospho-eIF2α, eIF2α, ATF4, and CHOP protein in HepG2 cells were detected using Western blotting. All experiments were repeated three times with similar results. **c** Densitometry analysis of the ratio of phosphorylated eIf2α to total eIf2α bands normalised to tubulin and relative to the corresponding control. Quantitative results of the phosphorylation of eIf2α are presented as the mean ± SD. **d** Real-time PCR analysis of the PERK targets in Huh7 and **e** Hep3B cells. *p < 0.05, **p < 0.01, ***p < 0.001

Because PlGF inhibition reduced tumour hypoxia and PERK activation *in vivo*, we questioned whether hypoxia mediates PERK activation in HCC cells. Therefore, we examined the effect of hypoxia (<1 % O_2_ or 7.6 mmHg [22]) for 24 h or 48 h on the expression of PERK targets in HepG2, Huh7 and Hep3B cells (Fig. 3a-b). Hypoxic exposure upregulated the mRNA expression of GRP78 (*p* < 0.001), ATF4 (*p* < 0.05), CHOP (*p* < 0.001) and GADD34 (*p* < 0.001). Furthermore, hypoxic exposure also increased the phosphorylation of eIF2α (24 h: *p* < 0.05 and 48 h: *p* < 0.01; Fig. 3b-c) and protein expression of ATF4 and CHOP (Fig. 3b). Accordingly, hypoxic exposure significantly upregulated CHOP and GADD34 mRNA in Huh7 and Hep3B cells (Fig. 3d-e). These data indicate that hypoxic exposure causes potent activation of the PERK pathway in HCC cells.

### Activation of the IRE1 pathway promotes PlGF expression

**Fig. 4:**
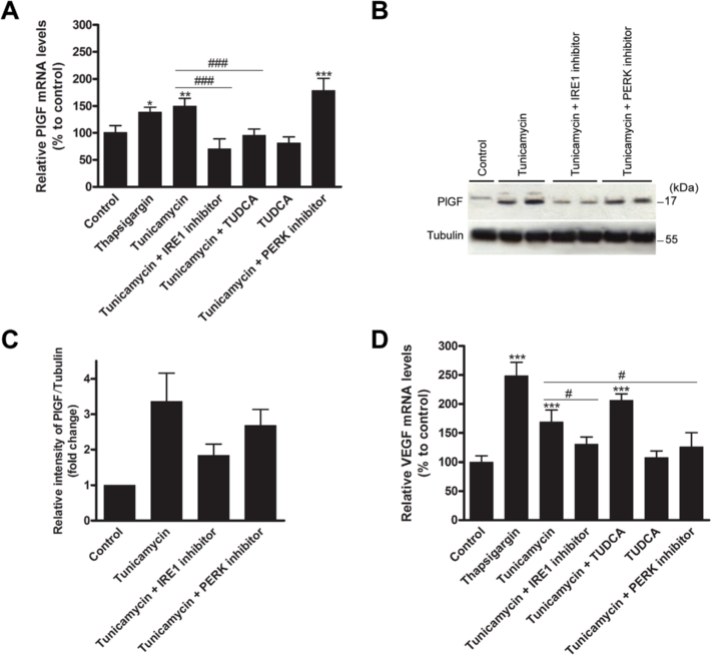
ER stress induces PlGF and VEGF expression in HepG2 cells. **a** Relative *PlGF* mRNA levels in HepG2 cells treated for 48 h with the indicated treatments. TUDCA: tauroursodeoxycholic acid. **b** Immunoblotting of cell lysates was performed to detect PlGF protein levels. All experiments were repeated three times with similar results. **c** Densitometry analysis of the PlGF bands normalized to tubulin and relative to the control. Quantitative results are presented as the mean ± SD. **d** Relative *VEGF* mRNA levels in HepG2 cells treated for 48 h with the indicated treatments. *p < 0.05, **p < 0.01, ***p < 0.001 compared to control. ^#^ *p* < 0.05, ^##^ *p* < 0.01, ^###^ *p* < 0.001 compared to the indicated group

Because the UPR is activated in HCC and PlGF inhibition is able to reduce activation of at least the Perk branch of the UPR, we next analysed the effect of ER stress on PlGF expression *in vitro*. Therefore, we used two different ER stress inducers: tunicamycin, an inhibitor of protein glycosylation, and thapsigargin, an inhibitor of sarcoplasmic/endoplasmic reticulum Ca^2+^ ATPases [13, 23]. Both significantly increased the mRNA levels of PlGF (Fig. 4a). As shown in Fig. 4b, an increase in the expression of faster-migrating unglycosylated PlGF was detected in HepG2 cells treated with tunicamycin. The addition of the chemical chaperone TUDCA to tunicamycin-treated cells attenuated the ER stress-mediated induction of PlGF mRNA (*p* < 0.01), whereas the addition of TUDCA to untreated cells had no effect on the PlGF mRNA levels (Fig. 4a). Addition of a small-molecule inhibitor of the IRE1 pathway reduced the tunicamycin-mediated upregulation of PlGF mRNA (*p* < 0.001, Fig. 4a) and protein (Fig. 4b-c) levels. In contrast, the addition of a small-molecule inhibitor of the PERK pathway did not affect PlGF expression. These data show that the ER stress-mediated upregulation of PlGF is primary regulated by the IRE1 pathway of the UPR. Finally, we assessed the effect of UPR activation on the expression of VEGF mRNA and observed that also VEGF was significantly upregulated by the ER stress inducers (*p* < 0.001, Fig. 4d). However, in contrast to PlGF mRNA, we observed that VEGF transcription is regulated by both the IRE1 and PERK pathway (*p* < 0.05) and is not attenuated by TUDCA.

## Discussion

Growing tumours are often subjected to deficiencies in vital nutrients and oxygen. These inadequate extracellular conditions can adversely affect the environment of the ER and impinge on the maturation of nascent proteins. We recently reported that PlGF inhibition induces vessel normalisation, potentially supporting the delivery of nutrients and oxygen to tumour cells [5, 24, 25].

**Fig. 5:**
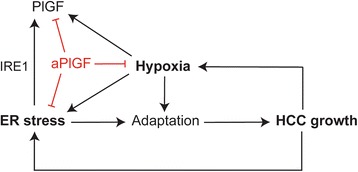
Schematic model outlining the interactions among PlGF, ER stress and hypoxia and their effects on HCC growth

In this study, we found that PlGF inhibition reduced intratumour hypoxia and ER stress levels (Fig. 5). In fact, PlGF inhibition attenuates the carcinogen-induced upregulation of chaperones, such as Grp78 and Grp94, and the activation of the Perk pathway without affecting Ire1 activation. These chaperones and Perk activation are pro-survival and pro-proliferative modulators in tumour cells [13–26]. Probably, the aPlGF-mediated reduction of these UPR factors tempers the aggressive growth of HCC cells.

Recently, hypoxia-inducible factor-1α (HIF-1α), a key transcription factor in the cellular response to hypoxia, was shown to be an important driver of HCC growth [27]. In this study, we showed that PlGF inhibition reduced tumour hypoxia and PERK activation *in vivo* and that hypoxia activates the PERK/phospho-IF2α/ATF4 cascade in HCC cells, suggesting that tumour hypoxia mediates the observed PERK activation in HCC. Possibly, tumour hypoxia is also involved in the pronounced activation of PERK in other tumour types, such as glioma [28]. Finally, because PERK is, next to hypoxia, able to stimulate tumour growth [13, 29], the normalisation of tumoural oxygen levels by PlGF inhibition is able to dually target pro-survival signalling via reduced activation of the HIF-1α and PERK pathway.

Because PlGF inhibition was previously reported to reduce experimental liver fibrosis [30], the contribution of hypoxia and ER stress modulation, which both have been implicated in fibrogenesis [31, 32], to this outcome requires further investigation.

Whereas the UPR has previously been shown to upregulate several angiogenic factors, including VEGF [33], this is, to our knowledge, the first report to demonstrate the induction of PlGF by ER stress in tumour cells. Because studies on transgenic mice have revealed that PlGF expression is restricted to pathological conditions [34], the further investigation of the role of ER stress in the selectivity of PlGF expression to pathological conditions, potentially characterised by ER stress, is indicated. Finally, the role of the UPR in vessel abnormalisation induced by excessive production of angiogenic factors requires further investigation [34].

Vice versa, the effect of therapies modulating tumour angiogenesis on the UPR activation pattern, which affects tumour growth, is currently unknown. To our knowledge, this is the first study to provide evidence that vessel normalisation regulates the UPR in cancer cells. We speculate that anti-VEGF therapies may exert their therapeutic effect in part by UPR modulation.

The promising preclinical findings of anti-PlGF in HCC but also in other tumour types such as medulloblastoma [35], together with the acceptable safety profile of anti-PlGF administration in Phase I clinical trials, have attracted attention to PlGF as a potential target for therapy. However, improved understanding of the effect on tumour biology is required. This study indicates that anti-PlGF modulates the tumour microenvironment and cell adaptation mechanisms, which have been linked to tumour behavior [13, 36].

## Conclusions

In summary, we have shown that inhibition of PlGF tempers UPR activation in HCC, most likely by improved oxygen delivery via the induced normalisation of tumour vessels. Moreover, we revealed that the UPR, in turn, regulates the expression of PlGF in HCC cells. Thus, our study sheds light on the reciprocal interactions between PlGF, hypoxia and the UPR and suggests that the antitumour effects of angiogenesis-modulating therapy could be mediated by modifying the tumour microenvironmental stresses in HCC.

## Additional files

Additional file 1:
**Supplementary methods.** Detailed information regarding total RNA extraction, quantitative real-time PCR, Western blotting, and immunohistochemistry. (DOCX 30 kb) Additional file 2: Figure S1. PlGF blockage induces vessel normalization. (A) Immunostaining for the endothelial marker endoglin (CD105). In HCC nodules, the capillary network is chaotically organized with tortuous vessels (indicated by red arrows) laying at large distances from each other. However, the capillaries in HCC after aPlGF treatment or in PlGFKO mice have a more normal appearance with regular pattern, size, and shape (indicated by green arrows). Black arrows and dashed lines indicate tumours. (B) Quantification of tortuous vessels per mm^2^; *n* = 5; **p* < 0.05, ***p* < 0.01. (TIFF 16370 kb) Additional file 3: Figure S2. Densitometry analysis of the phosphorylation of eIf2α in isolated HCC. (A) Densitometry analysis of the ratio of phosphorylated eIf2α to total eIf2α bands normalized to tubulin and relative to the corresponding control. Quantitative results of phosphorylation of eIf2α are presented as the mean ± SD. **p* < 0.05. (TIFF 4834 kb) Additional file 4: Figure S3. Effect of PlGF inhibition on the expression of the UPR sensor Perk. (A) Quantitative real-time PCR analysis of Perk. Relative fold changes were calculated using the ΔΔCT method. IgG = 25w DEN + 5w IgG, aPlGF = 25w DEN + 5w aPlGF, WT = 30w DEN in wild type (WT) mice, PlGFKO = 30w DEN in PlGF^−/−^ knockout mice. (B) Immunoblotting for Perk protein. (TIFF 8720 kb)

## Abbreviations

HCC: Hepatocellular carcinoma
VEGF: Vascular endothelial growth factor
PlGF: Placental growth factor
ER: Endoplasmic reticulum
PDIA4: Protein disulfide isomerase family A, member 4
CANX: Calnexin
GRP78: Glucose-regulated protein 78
GRP94: Glucose-regulated protein 94
UPR: Unfolded protein response
PERK: PKR-like endoplasmic reticulum kinase
IRE1: Inositol-requiring enzyme 1
ATF6: Activating transcription factor 6
eIF2α: Eukaryotic initiation factor 2α
ATF4: Activating transcription factor 4
CHOP: C/EBP homologous protein
XBP1: X-box-binding protein 1
XBP1s: Spliced XBP1
ERDJ4: Endoplasmic reticulum DnaJ homolog 4
XBP1u: Unspliced XBP1
HERPUD1: Homocysteine-responsive endoplasmic reticulum-resident ubiquitin-like domain member 1 protein
PlGFKO: PlGF knockout
DEN: Diethylnitrosamine
aPlGF: Anti-PlGF monoclonal antibody
TUDCA: Tauroursodeoxycholic acid
GADD34: Growth arrest and DNA damage-inducible protein
HIF-1α: Hypoxia-inducible factor-1α
