# Supplementary material for: Placental growth factor inhibition modulates the interplay between hypoxia and unfolded protein response in hepatocellular carcinoma
Source: BMC Cancer. 2016 Jan 11;16:9. doi: 10.1186/s12885-015-1990-6 (PMC4707726; doi:10.1186/s12885-015-1990-6)
Supplement: Additional file 1: — Supplementary methods. Detailed information regarding total RNA extraction, quantitative real-time PCR, Western blotting, and immunohistochemistry. (DOCX 30 kb) [file 12885_2015_1990_MOESM1_ESM.docx]

**Placental growth factor inhibition modulates the interplay between hypoxia and unfolded protein response in hepatocellular cancer**

1. **Supplementary Materials and Methods**

# Total RNA extraction

Total RNA was extracted from all samples using the RNeasy Mini Kit (Qiagen, Westburg BV, The Netherlands) with on-column DNAse treatment (Qiagen). Needle homogenization was performed. The purity and quantity of total RNA was assessed using spectrophotometry (Nanodrop; Thermo Scientific, Wilmington, USA). The ratio of absorption at 260 and 280 nm was used to define RNA purity; samples with a 260:280 ratio between 1.8 and 2.0 were accepted.

# Quantitative real-time PCR

One microgram of total RNA was converted into single-stranded cDNA by reverse transcription (iScript; Bio-Rad, California, USA) using oligo (dT) and random priming. The cDNA was diluted 1/10 and used for real-time quantification with SYBR Green (Sensimix; Bioline Reagents Ltd., London, UK) and 250 nM of each primer. A two-step program was run on a LightCyclerR 480 (Roche). Cycling conditions included 95°C for 10 minutes and 45 cycles of 95°C for 10 seconds followed by 1 cycle of 60°C for 1 minute. Melting curve analysis confirmed primer specificities. All reactions were performed in duplicate. Relative fold change was calculated using the ΔΔCT method instructed by Applied Biosystems. First, the normalization of the threshold cycle (CT) values of the target gene was performed with the CT of GAPDH in the same samples (ΔCT=CT target – CT GAPDH). The expression was normalized again with the control (ΔΔCT=ΔCT – ΔCT control), and the fold change was calculated (2^-ΔΔCT^). The PCR efficiency for each primer pair was calculated using a standard curve of reference cDNA. Amplification efficiency was determined using the formula 10^-1/slope^. The primer set sequences are listed in Table S1.

# Western blotting

Total protein extract was obtained by dissolving cells in RIPA buffer (1x PBS, 1% NP-40, 0.5% sodium deoxycholate, 0.1% SDS and 1× complete protease inhibitors (Roche Diagnostics)). The total protein yield was determined using Bradford reagent (Biorad). Approximately 25-50 µg of protein was loaded and separated by SDS-PAGE. The proteins were transferred to a PVDF membrane (Millipore), which was subsequently blocked and incubated with specific antibodies (Table S2) in 5% non-fat milk followed by horseradish peroxidase-conjugated secondary antibodies. UPR-related antibodies were validated using tunicamycin. ECL detection reagent (Amersham Life Science, New Jersey, USA) was used to visualize the specific proteins.

# Immunohistochemistry

Pimonidazole hydrochloride (Hypoxyprobe) was used as a hypoxia marker. Endoglin was used as an endothelial marker. Immunohistochemistry was performed on 5 μm sections of FFPE tissue by heat-induced epitope retrieval in 10 mM sodium citrate (pH=6) before blocking with 5% BSA-PBS. Samples were incubated overnight at 4°C with anti-pimonidazole antibody from the Hypoxyprobe-1 kit (1:200 dilution in PBS), anti-Endoglin or anti-phospho-eIf2α antibody (Table S2). EnVisionTM+ System-HRP (DAB) was applied as secondary antibody (Dako, Golstrup, Denmark), and sections were counterstained with hematoxylin. Staining was semi-quantitatively measured by 2 independent observers using Olympus Cell^D^ software.

## Supplementary Tables

**Table S1**. **Primers used for quantitative real-time PCR experiments**. The PCR efficiency of each primer pair was calculated using a standard curve of reference cDNA. Amplification efficiency (R²) was determined using the formula 10^-1/slope^.

| **Gene symbol** | **Reference sequence** | **Species** | **Forward primer** | **Reverse primer** | **Efficiency** | ***R*^2^** |
| --- | --- | --- | --- | --- | --- | --- |
| Gapdh | NM_008084.2 | Mus musculus | GCCGGCTCAGTGAGACAAG | TGGCACCTTCAGCAACAATG | 95.1 | 0.99 |
| Hmbs | NM_001110251.1 | Mus musculus | GTCCCTCGAACACGAGACGCT | GGCAGCTGCAAAGCTCTCTCCAT | 100.9 | 0.99 |
| Atf4 | NM_009716.2 | Mus musculus | GTTGAGCAGGAACGCAGTCTT | GGCAGAAGAGCACTGATCGTA | 96.2 | 0.98 |
| Chop | NM_007837.3 | Mus musculus | AGCGCAACATGACAGTGAAG | GTGTAATTCCAGGGGGAGGT | 101.6 | 0.99 |
| Xbp1u | NM_013842.2 | Mus musculus | TCTCAAGCCGCCCCTCCGTT | GTGGCTGGCGTGCAAGGGAT | 107.2 | 0.97 |
| Xbp1s | NM_013842.2 | Mus musculus | TCTCAAGCCGCCCCTCCGTT | CGGGGTTGCTGGTGTGCCAT | 97.6 | 0.98 |
| Pdia4 | NM_009787.2 | Mus musculus | ACGAGACCCCGGCGTTCGGA | TGGCACTTTGAGGAGGTGAGCC | 90.6 | 0.99 |
| Herpud1 | NM_022331.1 | Mus musculus | ACGCCAAGTGTCGTTGTGTGGTC | GCTCGACTGCGCTCAGGGATG | 92.8 | 0.99 |
| Erdj4 | NM_013760.4 | Mus musculus | CGCCCTGTGGCCCTGACTTG | AGCTTTCAGGGGCAAACAGCCA | 98.1 | 0.98 |
| Canx | NM_001110499.1 | Mus musculus | CAACAGGGGAGGTTTATTTTGCT | TCCCACTTTCCATCATATTTGGC | 101.2 | 0.99 |
| Grp94 | NM_011631.1 | Mus musculus | GAGGCGGCTCCTGAGACCGAA | GGACCCTCATGGTGCGTGGC | 101.1 | 0.98 |
| Grp78 | NM_001163434.1 | Mus musculus | TGCCGAGCTAAATTACACATTG | CCTTGTGGAGGGATGTACAGA | 107.2 | 0.99 |
| Perk | NM_010121.2 | Mus musculus | TGCCTGGCTCGAAGCACCAC | TGGTGCATCCATTGGGCTAGGA | 102 | 0.98 |
| Pfk | NM_001163487.1 | Mus musculus | GCCGGCTCAGTGAGACAAG | TGGCACCTTCAGCAACAATG | 95.1 | 0.98 |
| Glut1 | NM_011400.3 | Mus musculus | GCTTATGGGCTTCTCCAA ACT | GTGACACCTCTCCCACA TAC | 95.8 | 0.99 |
| Gadd34 | NM_008654.2 | Mus musculus | ACATGCGATATCCCGCGCGAC | CGATCGTGGGTCCGGACTGC | 96.5 | 0.99 |
| GAPDH | NM_001256799.1 | Homo sapiens | TGCACCACCAACTGCTTA GC | GGCATGGACTGTGGT CATGAG | 91.9 | 0.99 |
| HMBS | NM_000190.3 | Homo sapiens | GGCAATGCGGCTGCAA | GGGTACCCACGCGAATCAC | 101.2 | 0.99 |
| ATF4 | NM_001675.2 | Homo sapiens | GACCACGTTGGATGACACTTG | GGGAAGAGGTTGTAAGAAGGTG | 97.8 | 0.99 |
| CHOP | NM_001195053.1 | Homo sapiens | AAGGCACTGAGCGTATCATGT | TGAAGATACACTTCCTTCTTGAACA | 105.2 | 0.99 |
| XBP1u | NM_001079539.1 | Homo sapiens | AGACAGCGCTTGGGGATGGAT | CCTGCTGCAGAGGTGCACGTAG | 115.1 | 0.99 |
| XBP1s | NM_001079539.1 | Homo sapiens | AGACAGCGCTTGGGGATGGAT | CCTGCACCTGCTGCGGACTC | 110.2 | 0.99 |
| PDIA4 | NM_004911.4 | Homo sapiens | TCCCATTCCTGTTGCCAAGAT | GCCCTCGTAGTCTACAGCCT | 99.7 | 0.99 |
| GRP78 | NM_005347.4 | Homo sapiens | GGGAACGTCTGATTGGCGAT | CGTCAAAGACCGTGTTCTCG | 106.5 | 0.99 |
| HERPUD1 | NM_001010989.2 | Homo sapiens | TTCCAAAGCAGGAAAAACGGC | GCAGGCTCCTCTGTGGATTC | 93.7 | 0.96 |
| GADD34 | NM_014330.3 | Homo sapiens | TCCTCTGGCAATCCCCCATA | GGAACTGCTGGTTTTCAGCC | 109.1 | 0.98 |
| VEGF | NM_001025366.2 | Homo sapiens | TCCTCACACCATTGAAACCA | GATCCTGCCCTGTCTCTCTG | 103 | 0.98 |
| PlGF | NM_001207012.1 | Homo sapiens | CACCCGGCTCGTGTATTTAT | AGCAGGGAAACAGTTGGCTA | 103.2 | 0.97 |

**Table S2**. **Characteristics of the antibodies used in this study**. The specificity, isotype, clone number and catalog number of the antibodies are indicated. All antibodies were used according to the manufacturer’s instructions.

| **Antigen** | **Antibody isotype, clone** | **Company** | **Cat no.** |
| --- | --- | --- | --- |
| ATF4 | Rabbit polyclonal IgG | Santa Cruz | sc-200 |
| Phospho-eIF2α | Rabbit monoclonal IgG, 119A11 | Cell Signaling | 3597 |
| eIF2α | Rabbit polyclonal IgG | Cell Signaling | 9721 |
| CHOP | Mouse monoclonal IgG2a, L63F7 | Cell Signaling | 2895 |
| GADD34 | Rabbit polyclonal IgG, H193 | Santa Cruz | sc-8327 |
| GRP78 | Rabbit monoclonal IgG, C50B12 | Cell Signaling | 3177 |
| PERK | Rabbit polyclonal IgG | Sigma | P0073 |
| Pimonidazole | Mouse monoclonal IgG1, 4.3.11.3 | Hypoxyprobe | HP2-200 |
| Endoglin | Goat polyclonal IgG | R&D Systems | AF-1320 |
| PlGF | Goat polyclonal IgG | R&D Systems | AF-264-PB |
| Tubulin | Rabbit polyclonal IgG | Abcam | ab6046 |

## Supplementary Figure Legends

**Fig. S1. PlGF blockage induces vessel normalization.** (A) Immunostaining for the endothelial marker endoglin (CD105). In HCC nodules, the capillary network is chaotically organized with tortuous vessels (indicated by red arrows) laying at large distances from each other. However, the capillaries in HCC after aPlGF treatment or in PlGFKO mice have a more normal appearance with regular pattern, size, and shape (indicated by green arrows). Black arrows and dashed lines indicate tumours. (B) Quantification of tortuous vessels per mm^2^; n= 5; *p<0.05, **p<0.01.

**Fig. S2. Densitometry analysis of the phosphorylation of eIf2α in isolated HCC.** (A) Densitometry analysis of the ratio of phosphorylated eIf2α to total eIf2α bands normalized to tubulin and relative to the corresponding control. Quantitative results of phosphorylation of eIf2α are presented as the mean ± SD. *p<0.05.

**Fig. S3. Effect of PlGF inhibition on the expression of the UPR sensor Perk.** (A) Quantitative real-time PCR analysis of Perk. Relative fold changes were calculated using the ΔΔCT method. IgG= 25w DEN + 5w IgG, aPlGF= 25w DEN + 5w aPlGF, WT= 30w DEN in wild type (WT) mice, PlGFKO= 30w DEN in PlGF^-/-^ knockout mice. (B) Immunoblotting for Perk protein.
