# Supplementary figures and images for: Placental growth factor inhibition modulates the interplay between hypoxia and unfolded protein response in hepatocellular carcinoma
Source: BMC Cancer. 2016 Jan 11;16:9. doi: 10.1186/s12885-015-1990-6 (PMC4707726; doi:10.1186/s12885-015-1990-6)

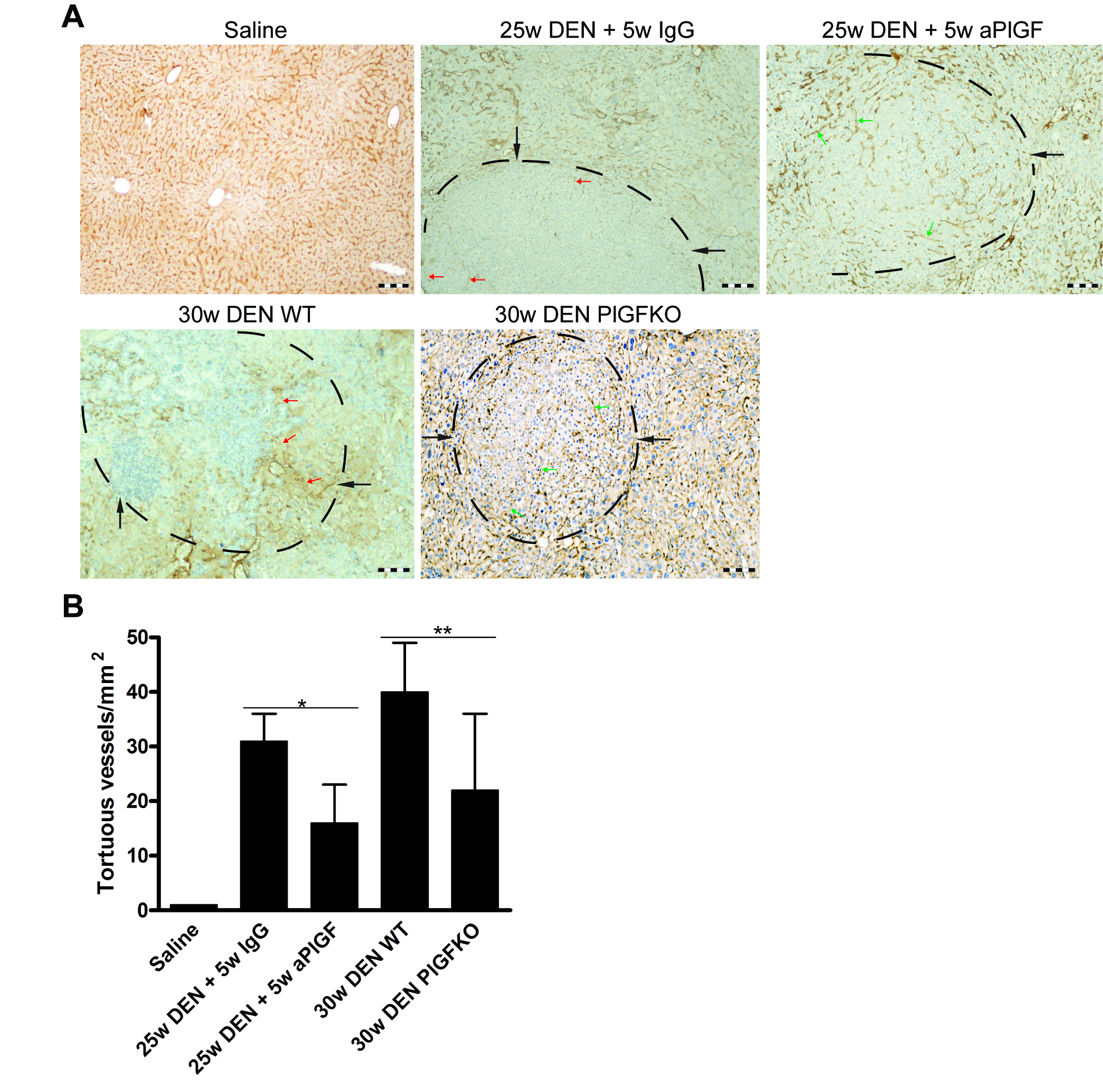

Supplement: Additional file 2: Figure S1. — PlGF blockage induces vessel normalization. (A) Immunostaining for the endothelial marker endoglin (CD105). In HCC nodules, the capillary network is chaotically organized with tortuous vessels (indicated by red arrows) laying at large distances from each other. However, the capillaries in HCC after aPlGF treatment or in PlGFKO mice have a more normal appearance with regular pattern, size, and shape (indicated by green arrows). Black arrows and dashed lines indicate tumours. (B) Quantification of tortuous vessels per mm2; n = 5; *p < 0.05, **p < 0.01. (TIFF 16370 kb) [file 12885_2015_1990_MOESM2_ESM.tiff]

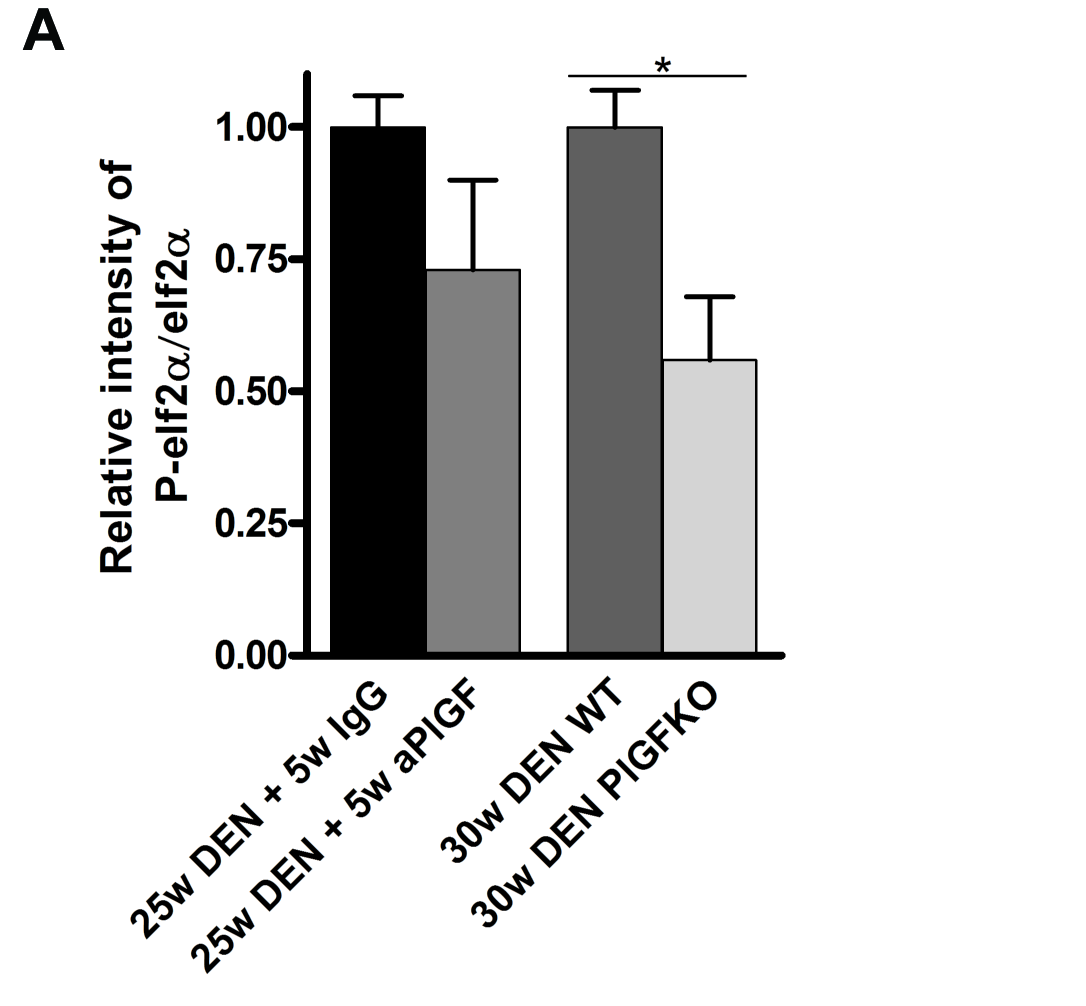

Supplement: Additional file 3: Figure S2. — Densitometry analysis of the phosphorylation of eIf2α in isolated HCC. (A) Densitometry analysis of the ratio of phosphorylated eIf2α to total eIf2α bands normalized to tubulin and relative to the corresponding control. Quantitative results of phosphorylation of eIf2α are presented as the mean ± SD. *p < 0.05. (TIFF 4834 kb) [file 12885_2015_1990_MOESM3_ESM.tiff]

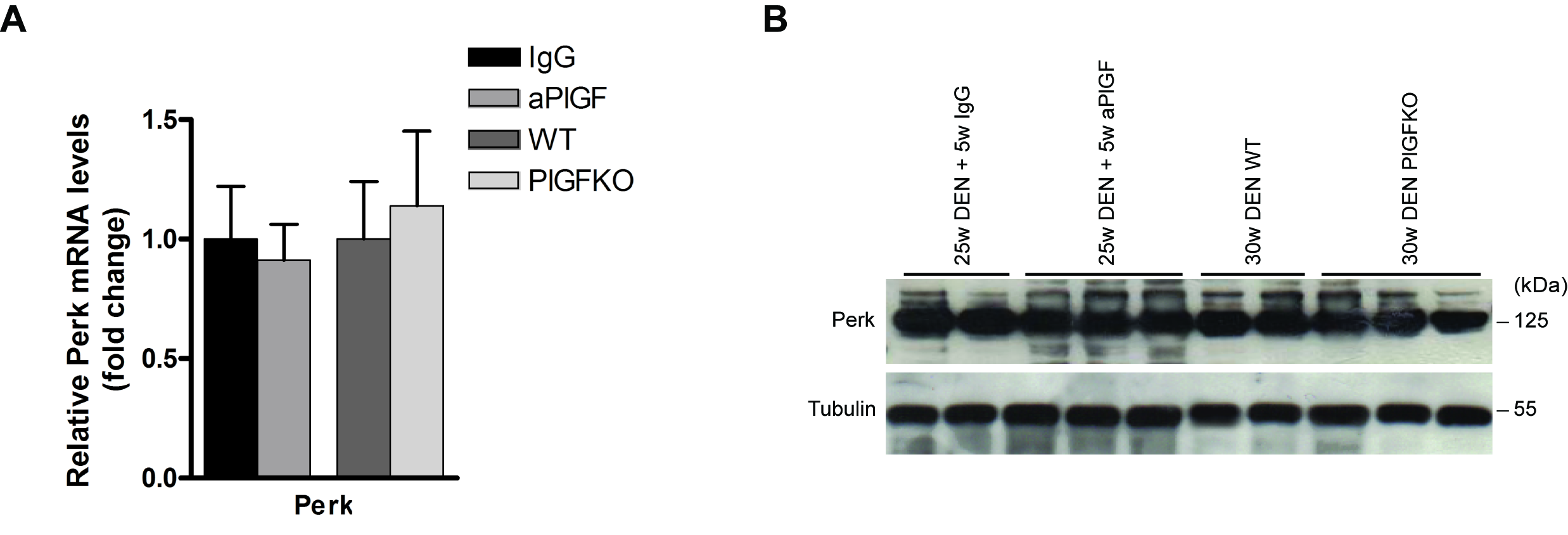

Supplement: Additional file 4: Figure S3. — Effect of PlGF inhibition on the expression of the UPR sensor Perk. (A) Quantitative real-time PCR analysis of Perk. Relative fold changes were calculated using the ΔΔCT method. IgG = 25w DEN + 5w IgG, aPlGF = 25w DEN + 5w aPlGF, WT = 30w DEN in wild type (WT) mice, PlGFKO = 30w DEN in PlGF−/− knockout mice. (B) Immunoblotting for Perk protein. (TIFF 8720 kb) [file 12885_2015_1990_MOESM4_ESM.tiff]
